# Protocol for selective DREADD-based chemogenetic inhibition of GABAergic amygdala neurons receiving hippocampal projections in rats

**DOI:** 10.1016/j.xpro.2025.104103

**Published:** 2025-09-24

**Authors:** Felice Cicciarelli, Giulia Concina, Luisella Milano, Annamaria Renna, Benedetto Sacchetti

**Affiliations:** 1 Rita Levi-Montalcini Department of Neuroscience, University of Turin, C.so Raffaello 30, 10125 Turin, Italy

**Keywords:** Cell Biology, Neuroscience, Structural Biology

## Abstract

Designer receptors exclusively activated by designer drugs (DREADDs) are a highly precise and effective method for manipulating neurons and other excitable cells. Here, we present an approach that combines viral anterograde transsynaptic transport with Gαi/o DREADD neuronal inhibition to control targeted neurons in a rat model. We describe how to implement this technique, from surgical the procedure to the behavioral assay. This protocol enables researchers to manipulate target neurons receiving input from distinct starter structures, thereby helping to unravel selective neuronal circuitry.

For complete details on the use and execution of this protocol, please refer to Concina et al.^1^

## Highlights

• Combinatorial AAV injections into the rat basolateral amygdala and ventral hippocampus
• Fear conditioning and remote fear memory segregation
• Behavioral assessment of chemogenetic manipulation of GABAergic neurons

## Abstract (editor highlights)

Publisher’s note: Undertaking any experimental protocol requires adherence to local institutional guidelines for laboratory safety and ethics.

## Graphical abstract

**Figure:**
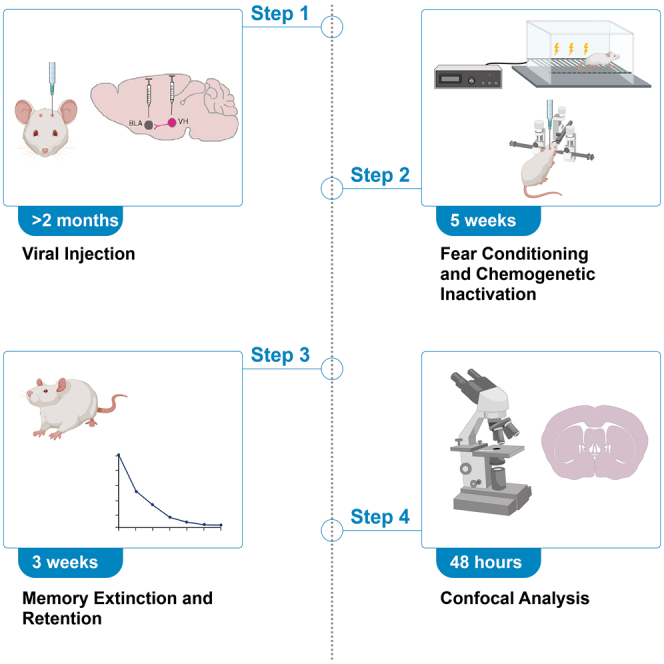

## Teaser

Designer receptors exclusively activated by designer drugs (DREADDs) are a highly precise and effective method for manipulating neurons and other excitable cells. Here, we present an approach that combines viral anterograde transsynaptic transport with Gαi/o DREADD neuronal inhibition to control targeted neurons in a rat model. We describe how to implement this technique, from the surgical procedure to the behavioral assay. This protocol enables researchers to manipulate target neurons receiving input from distinct starter structures, thereby helping to unravel selective neuronal circuitry.

## Before you begin

Chemogenetic technique allows researchers to manipulate excitable cells by controlling their activity. Along with optogenetic, this technique has been extremely valuable to dissect and comprehend neuronal circuits’ architecture, activity and regulation.^2^^,^^3^^,^^4^^,^^5^^,^^6^^,^^7^ The strength of these methods lies in the flexibility of the viral vectors which allow a plethora of manipulation strategies.

Among them, recombinant adeno associated viral vectors (rAAVs) are widely used in neuroscience because their tropism, transduction, and gene expression system can be easily modified.^8^

Several viral serotypes exhibit both anterograde and retrograde transduction capabilities, enabling the study of neuronal circuits among distant structures.^8^ An important advantage of rAAVs is the possibility to control gene expression through Cre-lox genetic switches, such as double-floxed inverted open-reading frame (DIO), or FLip-EXcision (FLEx) approaches.^8^ Combinatorial dual AAV vector techniques have been extensively used to decipher and manipulate neural circuits in the brain, and are based on two vectors, one expressing Cre recombinase and the other one expressing Cre-dependent transgene.^9^^,^^10^^,^^11^ The possibility to use also neuronal subtype-specific promoters allow researchers to study circuits in a very detailed way.^10^^,^^12^

This protocol describes how to identify and manipulate the anatomical and functional relationship between two selected brain structures, with a particular focus on the connections between a starter neural site and specific target neuronal cell types involved in defined behavioral or physiological functions. Specifically, this protocol focuses on the ability of hippocampal projecting neurons to regulate GABAergic neurons in the basolateral amygdala in rats during segregation of remote fear memories. These brain sites are selected as their well-defined role in the encoding of fear memories, together with other brain regions such as the sensory cortices.^13^^,^^14^^,^^15^^,^^16^

### Innovation

This protocol represents a significant innovation because it provides a precise method for manipulating the anatomical and functional connections between selected brain structures. Unlike approaches that study entire regions, this protocol focuses on analyzing subpopulation of neurons and how they influence a specific target cell type.

### Institutional permissions

All the experiments were approved by the Italian Ministry of Health (authorization no. 408/2020-PR) and by the local bioethical committee of the University of Turin.

### Animal preparation

**Timing: Months before the experiment**

1. House healthy male Sprague-Dawley rats (age, 65–70 days; weight, 240–350 g) in a plastic cage, 2–3 per cage, with food and water available ad libitum, under a 12 h light/dark cycle (lights on at 7:00 a.m.) at a constant temperature of 22 ± 1°C.

### Viral preparation

**Timing: At least a week before the experiment**

2. Use a trans-synaptic Cre virus AAV1-hSyn-Cre and Cre-dependent inhibitory DREADD virus AAV1-hDlx-DIO-KORD-mCyRFP (2x 10^13^ GC/mL and 4x 10^12^ GC/mL respectively).
3. Aliquot viral preparations in sterile Eppendorf tubes.
4. Store at −80°C until the day of the injection.

### Surgery setup preparation

**Timing: 1 h before surgery**

5. Sterilize surgical instruments and surfaces with 70% ethanol.
6. Prepare stereotaxic setup.
7. Thaw viral aliquots on ice.
8. Put a 10 μL Hamilton syringe with a 28-gauge needle mounted on an infusion pump.
9. Set the infusion rate between 0.1–0.3 μL/min.

## Key resources table

**Table:** 

REAGENT or RESOURCE	SOURCE	IDENTIFIER
**Antibodies**		

Guinea pig anti-RFP (working dilution 1:500)	Synaptic Systems	Cat# 390 005; RRID: AB_2737051
Rabbit anti-GAD65/67 (working dilution 1:1,000)	Abcam	Cat# ab49832; RRID: AB_880149
Cy3 anti-guinea pig (working dilution 1:1,000)	Jackson ImmunoResearch	Cat# 106-165-003; RRID: AB_2337423
Alexa Fluor 488 anti-rabbit (working dilution 1:1,000)	Invitrogen	Cat#A27034; RRID: AB_2536097
DAPI (working dilution 1:1,000)	Merck	Cat #D9542

**Bacterial and virus strains**		

AAV1-hSyn-Cre	Addgene	RRID: Addgene_105553
AAV1-hDlx-DIO-KORD-mCyRFP	UZH	N/A

**Chemicals, peptides, and recombinant proteins**		

Salvinorin B	Vinci-Biochem	CAY-23582-25
Paraformaldehyde	EMS	30525-89-4
Isoflurane	Sigma	792632
Ketoprofen	N/A	N/A
Sucrose	Sigma	S7903
PBS	Sigma	P4417
Saline (NaCl)	Sigma	746398

**Experimental models: Organisms/strains**		

Adult male wild-type Sprague-Dawley rats (age, 65–70 days; weight, 240–350 g)	Internal facility	N/A

**Software and algorithms**		

ImageJ	NIH	https://imagej.net
OBS Studio	OBS	https://obsproject.com/
G∗Power	NA	https://www.psychologie.hhu.de/arbeitsgruppen/allgemeine-psychologie-und-arbeitspsychologie/gpower
SPSS 22	IBM	N/A

**Other**		

Lab standard stereotaxic instrument	Stoelting	51600
Fear conditioning apparatus Habitest	Coulbourn	N/A
Microdrill	RWD	78001
0.6 mm tips	RWD	78001
Scalpel blades	FST	10020-00
Clamps	FST	13010-12
Forceps	FST	91113-10
Surgical stitches	FST	12040-02
Hamilton syringe with 28-gauge needle	Hamilton	80383
Infusion tubes	FST	N/A
Eppendorf tubes 0.5 mL	Sigma	EP0030108035
USB recording camera	Sony	N/A
Cryostat	Leica	CM1850
Confocal microscope	Zeiss	LSM800
Syringe pump	Stoelting	53129

## Step-by-step method details

### Viral injection into the ventral hippocampus and BLA

**Timing: 50 min for each rat**

To assess whether axons from hippocampal excitatory neurons that form synapses with neurons in the BLA are involved in a specific behavior, a trans-synaptic AAV1-hSyn-Cre will be injected into the ventral hippocampus, while a Cre-dependent inhibitory DREADD (AAV1-hDlx-DIO-KORD-mCyRFP) will be delivered into the BLA.

**Table:** 

Reagent	Final concentration
AAV1-hSyn-Cre	2x 10^13^ GC/mL
AAV1-hDlx-DIO-KORD-mCyRFP	4x 10^12^ GC/mL

1. Place the animal in the induction chamber.
  a. Start isoflurane flow at 4% and turn the oxygen flow meter to 2 L/min rate.
  b. Assess loss of consciousness of the animal via tail/toe pinch.
  c. Transfer the animal to the surgical surface.
2. Switch the anesthesia tubing to the nose cone.
  a. Place the animal’s nose in the nose cone and decrease the isoflurane concentration to 2%.
3. Fill the infusion tube with AAV1-hSyn-Cre suspension and expel a drop of solution to ensure clogs or trapped air in the tube/needle.
4. Position the animal in the stereotaxic frame by adjusting the ear-bars.
  a. Make sure to not damage eardrums and that there are no signs of head movements.
  b. Clean the head of the animal with 70% ethanol and remove the fur in the area of interest.
  c. Make a rostro-caudal incision on the scalp along the midline using a scalpel blade.
  d. Use a pair of clamps to expose the skull by gently opening the cut.
  e. Clean the bone with a dry cotton swab.
5. Calibrate the stereotaxic coordinate system.
  a. Find the bregma position, lower the syringe tip until it touches the bone.
  b. Reset all the stereotaxic coordinates.
  c. Retract the syringe from the skull.
  d. Move the syringe to the lambda and lower the syringe until it touches the bone.
  e. Check that the DV value is 0 ± 10 μm.
6. Retract the syringe needle from the skull once the system is calibrated and move the stereotaxic arm to the target coordinates.
  ***Note:*** Use two stereotaxic coordinates, an anterior and a posterior one, to effectively reach the entire ventral hippocampus.
  a. Ventral Hippocampus: AP: −5.0 L: ±5.0 DV: −6.0 and AP: −6.0 L: ±5.0 DV: −6.0.^17^
  b. Execute craniotomies at the selected coordinates using a microdrill equipped with 0.6 mm diameter tips.
  c. Perform a single hole for each injection site.
  d. Slowly and carefully lower the needle until it reaches the right DV coordinate.
  e. Inject 1 μL of the viral solution between 0.1–0.3 μL/min rate.
  f. Wait 5 min before retracting the needle.
  g. Slowly retract the syringe.
  h. Check for clog formation in the needle by spilling out ∼10 nL of solution.
7. Repeat step 6 for the other hemisphere and for all the other stereotaxic coordinates referred to the ventral hippocampus.
8. Remove the syringe and the tube from the stereotaxic arm and replace it with clean ones.
9. Fill new infusion tube with AAV1-hDlx-DIO-KORD-mCyRFP suspension and expel a drop of solution to ensure clogs or trapped air in the tube/needle.
10. Repeat steps 5–6 for the BLA coordinates and inject 0.8 μL per site.
11. Pull the skin together by using forceps and apply sterile surgical stitches to seal the incision.
12. Perform subcutaneous injection of analgesic/anti-inflammatory ketoprofen 2 mg/kg body weight.
13. Place the animal back in its home cage kept warm and under observation until full recovery.
14. Return the rat to its housing environment.
15. House animals for 5 weeks to allow recombination in the BLA and sufficient expression of DREADDs receptors.

### Auditory fear conditioning

**Timing: 5 min (for steps 16–19)**

**Timing: 6 h of waiting between steps 16–19 and 20–23**

**Timing: 5 min (for steps 20–23)**

Prior to the chemogenetic manipulation, rats learn two distinct fear events occurring in close temporal proximity.

16. Gently take an animal from its home cage to the soundproof room.
17. Place it inside the conditioning apparatus consisting of a rectangular black cage (32 x 40 x 30 cm) equipped with a stainless-steel rods grid (1 cm in diameter, spaced 1.5 cm apart) connected to a shock delivery set-up.
18. Let the animal freely explore the cage for 2 min. Initiate the fear conditioning where a pure tone of 15 kHz of frequency (named conditioned stimulus CS1, 15 sec, 80 dB, 36 sec inter-trials interval ITI) is paired seven times with a 1 sec mild foot shock (0.5 mA, unconditioned stimulus, US).
19. Return the animal back to its home cage after the procedure.
20. Take the same animal again to the soundproof room 6 hours after the first conditioning procedure,
  a. Use a different transport box to further differentiate the experiences.
21. Place the animal inside a different conditioning apparatus to further differentiate the two events.
22. Initiate the fear conditioning where a pure tone of 1 kHz of frequency (named conditioned stimulus CS2, 8 sec, 80 dB, 22 sec ITI) is paired seven times with the same US as the CS1.
23. Return the animal back to its home cage after the procedure.

### Chemogenetic blockade

This paragraph shows the procedure to inhibit BLA GABAergic neurons receiving projections from the ventral hippocampus in order to assess their involvement during specific behavior. Here is illustrated the role of these cells in the segregation of emotional memories as described in Concina et al.^1^

**Table:** 

Reagent	Final concentration
Salvinorin B	3 μM
Saline (NaCl)	0.9%

#### Salvinorin B infusion into the BLA

**Timing: 30 min**

24. Prepare the Salvinorin B (SalB) solution to induce chemogenetic blockade of infected cells inside the BLA after the second fear conditioning session.
  a. Dissolve SalB in sterile 0.9% saline solution at the final concentration of 3 μM.
  b. Mix well until no precipitates are present in the solution.
  c. Store the SalB solution at room temperature preventing light exposure.
25. Prepare the surgical room as described in the “Surgery setup preparation”.
26. Transport the animal to the surgical room 90 minutes after the end of the CS2-US pairing learning procedure.

***Note:*** Use the same transport cage after the second fear learning procedure.

27. Anesthetize the animal as reported in the paragraph “Viral injection into the ventral hippocampus and BLA” steps 1–2.
28. Fill the infusion tube with SalB taking care of clogs or air bubbles as previously described.
29. Position the animal in the stereotaxic frame by adjusting the ear-bars.
  a. Make sure to not damage eardrums and that there are no signs of head movements.
  b. Clean the old scar of the animal with 70% ethanol and carefully remove the stitches.
  c. Make a rostro-caudal incision on the scalp along the midline using a scalpel blade.
  d. Use a pair of clamps to expose the skull by gently opening the cut.
  e. Clean the bone with cotton swab.
30. Calibrate the stereotaxic coordinate system as described in step 5.
31. Move the stereotaxic arm to the BLA coordinates AP: −2.8 L: +5.5 and slowly lower the needle until it reaches DV: −8.0.
  a. Inject 0.5 μL of SalB between 0.1–0.3 μL/min flow rate.
  b. Wait 3 min before retracting the needle.
  c. Slowly retract the syringe.
  d. Check for clog formation in the needle by spilling out 10 nL of solution.
32. Repeat step 30 for the other hemisphere.
33. Follow steps 11–14 to finish the surgical procedure.

### Behavioral assay

This paragraph shows the procedure to assess the behavioral outcome of the chemogenetic blockade of BLA GABAergic neurons activated by hippocampal neurons. In the first part animals perform the extinction of the CS1 memory. In the second part the CS2 memory retention test.

#### CS1 memory extinction

**Timing: 35 min**

34. Transport singularly the animal from the facility to the behavioral rooms 3 weeks after the SalB treatment.
  a. Place the animal in the test cage (plastic walls and floor, 45 x 30 x 20 cm) inside a sound-attenuating box equipped with an exhaust fan, which eliminated odorized air from the enclosure and provided background noise of 60 dB and a video camera.
  b. Let the animal explore the cage for 2 min.
  c. Present to the animal the CS1 35 times (pure tone of 15-kHz, 15 s, 80 dB, 50 s ITI) in the absence of the US, for a total duration of 30 min.
  d. Record the behavior of the rat by using OBS video recording software.
  e. Return the animal back to its home cage.
  f. Clean the cage with 70% ethanol solution after each test.

#### CS2 memory retention

**Timing: 5 min**

35. Place the animal in the same cage the following day and let it explore for 2 minutes.
36. Assess CS2 memory retention by presenting the CS2 (pure tone of 1-kHz, 8 s, 80 dB, 22 s ITI) 7 times in the absence of the US.
37. Record the behavior of the rat by using OBS video recording software.
38. Return the animal back to its home cage.

### Tissue collection and preparation

**Table:** 

Reagent	Final concentration
Paraformaldehyde	4%
PBS	0,12 M
Sucrose	30%
anti-RFP	1:500
anti GAD65/67	1:1000
Cy3 anti-guinea pig	1:1000
Alexa Fluor 488 anti-rabbit	1:1000
DAPI	1:1000

#### Perfusion, tissue collection, and storage

**Timing: 20 min (for steps 39–49)**

**Timing: 2 days (for steps 50–52)**

39. Transport the animal to the perfusion room after completing the behavioral procedure.
40. Anesthetize the animal by placing it in the induction chamber using 4% isoflurane and 95% O_2_ at a flow rate of 2.0 L/min.
41. Switch the isoflurane from the chamber to a nosecone.
42. Place the rat to the perfusion station and assess reflexes to ensure anesthesia.
43. Secure the animal to the surface.
44. Grab the sternum with forceps and open the thoracic cavity to expose the heart.
45. Insert a needle into the left ventricle and activate the peristaltic pump to initiate the perfusion.
  a. Perfuse with PBS for 1 min to remove blood.
  b. Switch to 4% Paraformaldehyde (PFA) solution diluted in PBS and perfuse with 200 mL.
46. Stop the peristaltic pump and remove the needle from the animal heart.
47. Decapitate the animal.
48. Gently dissect the brain.
49. Place it in 4% PFA solution for 24 h at 4°C.
50. Remove the PFA solution the following day.
51. Transfer the brain into a 30% Sucrose solution.
52. Place the brain at 4°C for two days.

#### Tissue slicing and immunofluorescence

**Timing: 2 days**

**Table:** 

Reagent	Final concentration
PBS	0,12 M
anti-RFP	1:500
anti GAD65/67	1:1000
Cy3 anti-guinea pig	1:1000
Alexa Fluor 488 anti-rabbit	1:1000
DAPI	1:1000

53. Place the brain in a PBS solution and remove the cerebellum.
54. Mount the brain on a Cryostat and section at 35 μm thickness.
55. Incubate brain slices by using primary polyclonal guinea pig anti-RFP (1:500, SySy) and rabbit anti GAD65/67 (1:1000 dilution, Abcam).
56. Incubate slices the following day for 1 h at RT with secondary fluorescent Cy3 anti-guinea pig (1:1000, Jackson), Alexa Fluor 488 anti-rabbit (1:1000, Invitrogen).
57. Stain cell nuclei with DAPI.

### Tissue imaging and analysis

#### Confocal analysis

**Timing: Variable**

58. Capture BLA images using a confocal microscope (Zeiss Airyscan) at the AP coordinates from 2.4 to 3.5 mm from the bregma.
59. Use three lasers (405, 488 and 568 nm), each corresponding to the peak emission spectrum for DAPI (Nissl stain for cell nuclei), Alexa Fluor 488 and mCyRFP, respectively.
60. Acquire images with 40X objective using 15 sections stack spaced 1.5 μm apart.
61. Analyze the images by using the ImageJ software.
62. Count the number of KORD-expressing cells (mCyRFP) and GAD65/67-expressing cells (Alexa 488).
63. Normalize cell counts to the total number of DAPI cells.

## Expected outcomes

This protocol allows specific manipulation of projected neurons from a determined anatomical area to a target one. Concina et al. exploited this technique to selectively manipulate GABAergic neurons in the BLA. However, by using different viral combinations, it is possible to investigate the role of other neuronal subpopulations or neural circuits. This approach allows researchers to address two key questions: which subtypes of neurons in the BLA receive inputs from the ventral hippocampus, and what is their role in the segregation of remote auditory fear memories?

Chemogenetic inactivation of BLA neurons shows the implications of these cells in the separation of these memories.

**Figure 1:**
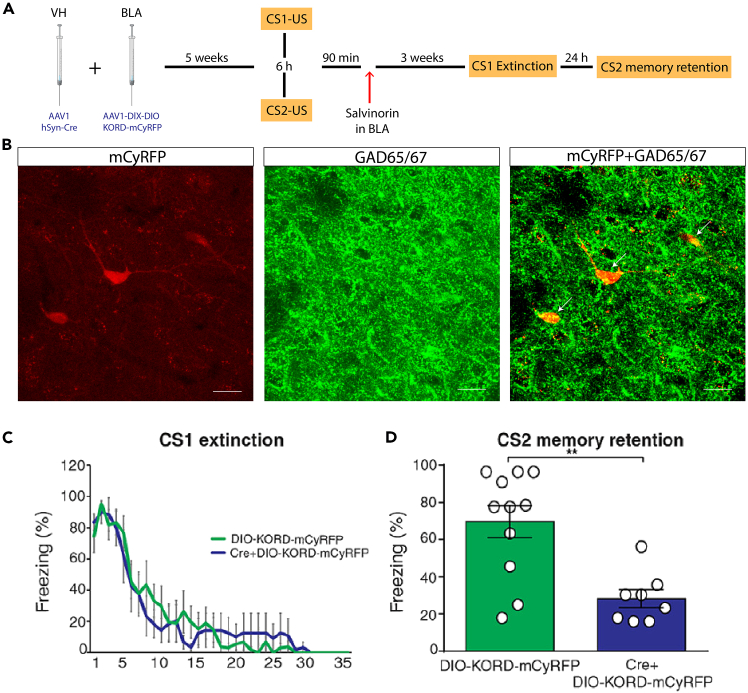
Expected results (A) Experimental design. (B) Confocal images that illustrate colocalization of KORD-positive cells (mCyRFP) and inhibitory neurons (GAD65/67). Scale bar 20 μm. (C) Curves of CS1 memory extinction of AAV1-hSyn-Cre + AAV1-hDlx-DIO-KORD-mCyRFP injected rats and AAV1-hDlx-DIO-KORD-mCyRFP injected rats. (D) Freezing time to CS2 after CS1 extinction in the two experimental groups. Data are means ± SEM. ∗*p* < 0.05 and ∗∗*p* < 0.01.

Confocal images confirm colocalization of viral gene reporter mCyRFP, thus KORD-positive, with GAD65/67, a specific marker of inhibitory neurons (Figure 1A).

## Quantification and statistical analysis

1. Statistical analysis.
  a. Use SPSS Statistics 22 (IBM) for statistical analysis and graphical representation of data.
  b. Use G∗power software to estimate the sample size for each group based on effect size calculation.
  c. Determine normal distribution by Kolmogorov-Smirnov test.
  d. Compare normally distributed data from two groups with two-tailed unpaired Student’s t-tests.
  e. Determine whether the data met the assumptions of the statistical approach: the null hypothesis was rejected at *p* < 0.05 (∗*p* < 0.05, ∗∗*p* < 0.01, ∗∗∗*p* < 0.001 and ∗∗∗∗*p* < 0.0001).
  f. Present data as mean ± SEM.

## Limitations

This protocol gives an effective way to analyze the role of a specific subpopulation of neurons in a function/behavior of interest.

Chemogenetic and optogenetic approaches are to date the best strategies to study functional targeted activation/inactivation of neurons, and KORD system has been shown to be effective.^18^

Chemogenetic still remains an experimental manipulation of these cells which could not represent exactly a natural outcome.

Furthermore, behavioral experiments are subjected to a certain level of variability, and this could need a higher sample size to study specific pathways.

The protocol itself relies on viral infection and recombination, thus it could have some limitations in the range of infected cells, because two events need to happen.

## Troubleshooting

### Problem 1

Absence of KORD-positive cells (step 61).

### Potential solution 1

The expression of KORD-mCyRFP genes depends on the recombination of the two viral vectors. In addition, the AAV1-hSyn-Cre virus needs to migrate along the projecting axons.

Try to increase the infection time, a minimum of 5 weeks is needed for adult male rats (age, 65–70 days; weight, 240–350 g).

### Potential solution 2

Flow rate and volume of viral solution can influence the results of the infection and recombination. Try to decrease the injection speed or to modify the volume depending on the model used. Eventually consider using a nanoinjector as a delivery method.

### Potential solution 3

Exclude animals with inadequate expression from analysis.

### Problem 2

Chemogenetic manipulation can trigger off-target behavioral response.

### Potential solution 1

Before including animals in the analysis verify the injection site and the extension of the infection. It is fundamental to include only animals with KORD-positive cells inside the BLA.

### Potential solution 2

To detect non-specific effect of SalB injection in the BLA, a control group injected only with AAV1-hDlx-DIO-KORD-mCyRFP treated with SalB should be included in the analysis (Figure 1B).

### Problem 3

Behavioral effects of chemogenetic blockade may not be clearly noticeable in some rats.

### Potential solution

Verify the presence of KORD-positive cells throughout the whole anatomical structure and in both the hemispheres.

## Resource availability

### Lead contact

Further information and requests for resources and reagents should be directed to and will be fulfilled by the lead contact, Benedetto Sacchetti (benedetto.sacchetti@unito.it).

### Technical contact

Questions about the technical specifics of the protocol should be directed to the technical contact, Felice Cicciarelli (felice.cicciarelli@unito.it).

### Materials availability

This study did not generate new unique reagents.

### Data and code availability

This study did not generate new code.

## Acknowledgments

This work was supported by Fondazione Cariverona (no. 2018.0780), CRT (2021.0485), Banca d’Italia (D63C23000630005), and the grant ‘‘Progetti di ricerca di Rilevante Interesse Nazionale (PRIN)’’ (20178NNRCR_002 and 2022BYZPFE) from the Italian Ministry of University and Research (MIUR).

## Author contributions

G.C., F.C., and B.S. wrote the manuscript. A.R. and L.M. generated the figures and protocols. All authors discussed the results and commented on the manuscript.

## Declaration of interests

The authors declare no competing interests.
